# Silk fibroin vascular graft: a promising tissue-engineered scaffold material for abdominal venous system replacement

**DOI:** 10.1038/s41598-020-78020-y

**Published:** 2020-12-03

**Authors:** Sho Kiritani, Junichi Kaneko, Daisuke Ito, Masaaki Morito, Takeaki Ishizawa, Nobuhisa Akamatsu, Mariko Tanaka, Takuya Iida, Takashi Tanaka, Ryo Tanaka, Tetsuo Asakura, Junichi Arita, Kiyoshi Hasegawa

**Affiliations:** 1 Hepato-Biliary-Pancreatic Surgery Division, Department of Surgery, Graduate School of Medicine, The University of Tokyo, 7-3-1 Hongo, Bunkyo-ku, Tokyo, 113-8655 Japan; 2 Department of Pathology, Graduate School of Medicine, The University of Tokyo, Tokyo, Japan; 3 Department of Plastic and Reconstructive Surgery, Graduate School of Medicine, The University of Tokyo, Tokyo, Japan; 4 Department of Veterinary Surgery, Tokyo University of Agriculture and Technology, Tokyo, Japan; 5 Department of Biotechnology, Tokyo University of Agriculture and Technology, Tokyo, Japan

**Keywords:** Translational research, Gastroenterology

## Abstract

No alternative tissue-engineered vascular grafts for the abdominal venous system are reported. The present study focused on the development of new tissue-engineered vascular graft using a silk-based scaffold material for abdominal venous system replacement. A rat vein, the inferior vena cava, was replaced by a silk fibroin (SF, a biocompatible natural insoluble protein present in silk thread), tissue-engineered vascular graft (10 mm long, 3 mm diameter, n = 19, SF group). The 1 and 4 -week patency rates and histologic reactions were compared with those of expanded polytetrafluoroethylene vascular grafts (n = 10, ePTFE group). The patency rate at 1 and 4 weeks after replacement in the SF group was 100.0% and 94.7%, and that in the ePTFE group was 100.0% and 80.0%, respectively. There was no significant difference between groups (*p* = 0.36). Unlike the ePTFE graft, CD31-positive endothelial cells covered the whole luminal surface of the SF vascular graft at 4 weeks, indicating better endothelialization. SF vascular grafts may be a promising tissue-engineered scaffold material for abdominal venous system replacement.

## Introduction

An R0 resection, in which the resection margin is free of cancer cells microscopically, is the only curative treatment for hepato-biliary-pancreatic malignant tumors, such as hepatocellular carcinoma, colorectal liver metastasis, biliary tract cancer, and pancreatic cancer. These types of malignant tumors often invade the nearby major abdominal venous system, including the hepatic vein^1,2^, inferior vena cava (IVC)^3^, portal vein, and superior mesenteric vein^4,5^, leading surgeons to attempt precise and extensive resection of the malignant tumor including the abdominal venous system to achieve an R0 resection. These surgical procedures depend on the ability of the surgeon to obtain a size-matched graft to replace the venous defect.

Unlike arterial replacement, venous replacement is rarely reported and there are several inherent limitations. The use of tailor-made autologous venous grafts utilizing the iliac vein^6^, great saphenous vein^7^, renal vein^8^, and internal jugular vein^9^ has been reported, but smaller graft size, additional surgical incisions, and longer operation time are disadvantages of autologous venous grafts. Although cryopreserved homologous veins produce better results^10^, the donor shortage and higher preservation cost remain problematic. Synthetic vascular grafts, expanded polytetrafluoroethylene (ePTFE) and polyethylene terephthalate, are used mainly for arterial replacement and their application for reconstruction of the venous system has been attempted^11^. In hepato-biliary-pancreatic surgery, however, surgeons are reluctant to use artificial vascular grafts because of low-flow thrombogenicity without endothelialization and higher graft infection rates in contaminated tissue beds under digestive fluid^12^. Furthermore, these synthetic vascular grafts permanently remain in the human body and the long-term outcome is unclear. To date, no alternative tissue-engineered venous grafts have been reported. A new tissue-engineered venous graft with better endothelialization and a more tolerable risk of infection using absorbable material is desirable.

Silk fiber is a natural protein fiber and silk thread has long been used in surgery for suturing and ligature^13^. Silk fibers comprise silk fibroin (SF) and silk sericin^14^. Silk sericin is an antigenic gum-like protein that surrounds the SF core fibers^13^ and can be removed through a degumming process^15^. SF biomaterial has biologic advantages, such as better biocompatibility, high affinity for cells, and susceptibility to proteolytic degradation in vivo without antigenicity^16,17^. Recently, experimental artery replacement using double-raschel knitted SF vascular grafts coated with an SF sponge was reported in animal models^18,19^. Enomoto et al. first reported rat aorta replacement using an SF graft with a 1-year patency rate of 85%. Anti-CD31 and anti-smooth muscle actin immunostaining revealed that endothelial cells and smooth muscle cells migrate into the SF graft early after implantation and become organized into endothelial and medial layers^18^. No venous replacement model has yet been reported.

We evaluated the patency rate of double-raschel knitted SF grafts coated with an SF sponge as an abdominal venous system replacement for the IVC in a rat model. In addition, we investigated the histologic reaction to the SF graft. We hypothesized that SF grafts would have a better patency rate than ePTFE grafts.

## Results

### Scanning electron microscopy observation and mechanical properties of SF grafts

**Figure 1:**
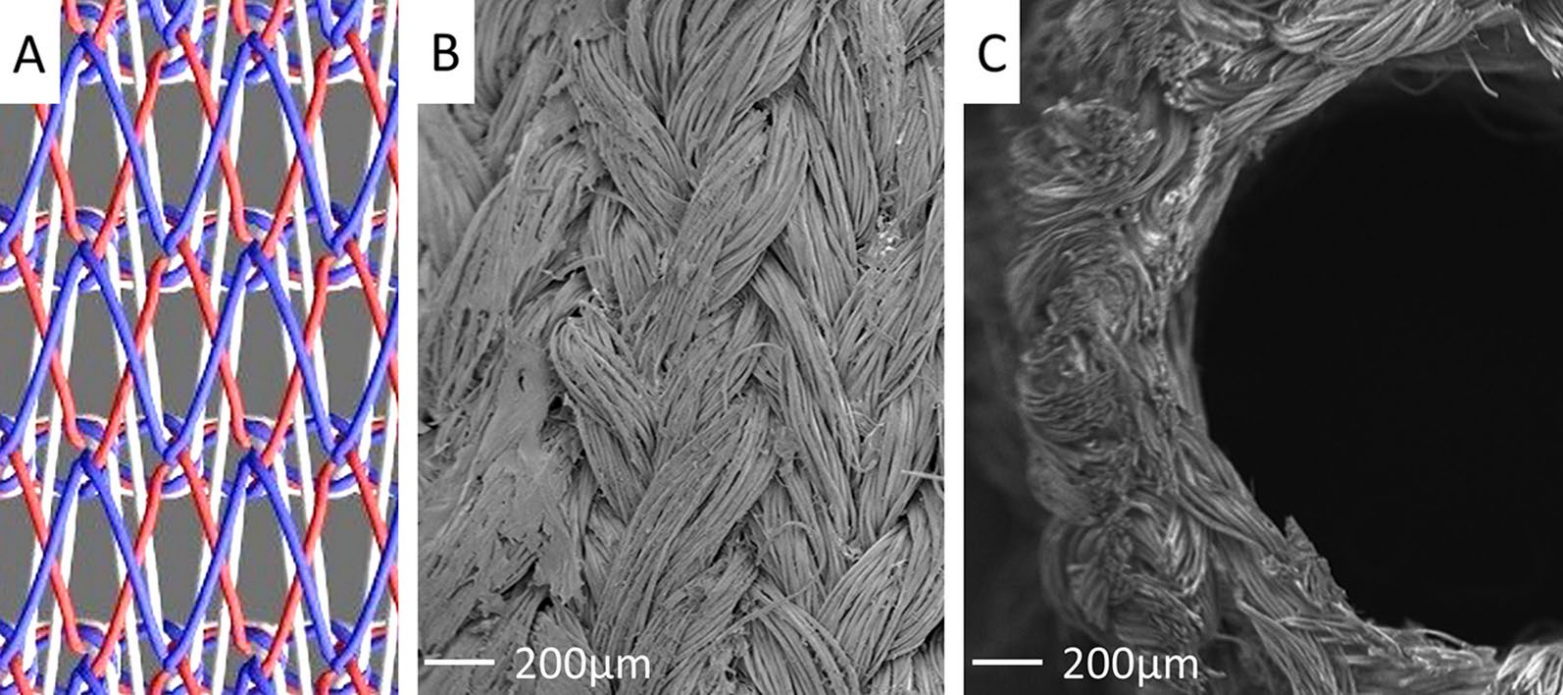
A schematic figure of the double-raschel knitting pattern (**A**). Scanning electron microscopy images showed the surfaces (**B**) and cross-sections (**C**) of SF grafts coated with SF sponges. Images were processed by a software, Microsoft PowerPoint Ver. 365, https://www.microsoft.com/ja-jp/education/products/office.

A schematic figure of the double-raschel knitting pattern is shown in Fig. 1A. Scanning electron microscopy images, including the surfaces and cross-sections of SF grafts coated with SF sponges are shown in Fig. 1B,C. The longitudinal suture retention strength, circumferential tensile strength, and circumferential compressive elastic modulus of SF grafts coated with SF sponges were 6.4 ± 0.6 N, 51.0 ± 3.0 N, and 0.013 ± 0.002 N mm^2^, respectively. Porosity was 82%.

### Animal model

**Figure 2:**
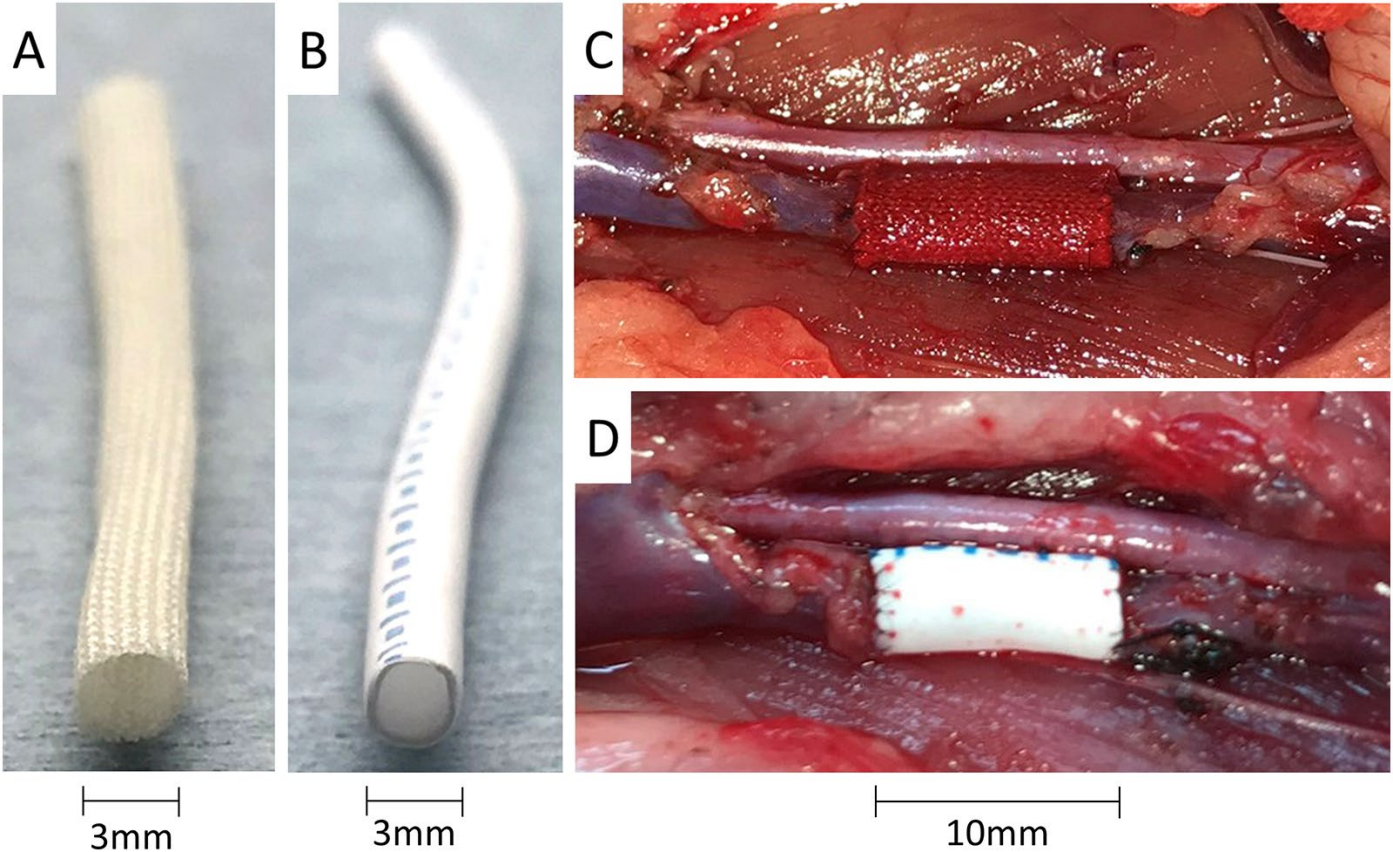
Photo shows an SF vascular graft (**A**) and an ePTFE vascular graft (**B**). Gross view of the SF graft (**C**) and ePTFE graft (**D**) used to replace a rat IVC. The SF graft quickly absorbed blood after recanalization (**C**). ePTFE; Expanded polytetrafluoroethylene, SF; silk fibroin. Images were processed by a software, Microsoft PowerPoint Ver. 365, https://www.microsoft.com/ja-jp/education/products/office.

The number of rats, age, and mean body weight were as follows; SF group: n = 19, 13 weeks, 450 g, and the ePTFE group: n = 10, 12 weeks, 405 g, respectively. The surgical time did not differ significantly between groups (SF group, 43 min vs ePTFE group, 48 min; Table 1). The SF graft was soft and flexible, similar to the ePTFE graft. An SF vascular graft before and immediate after IVC replacement shown in Fig. 2A,C. The SF graft quickly absorbed blood after recanalization and became reddish in color without bleeding. The ePTFE graft was still whitish in color after recanalization (Fig. 2B,D). No rats died before we evaluated the graft patency.

**Table 1:** Animal model.

	SF (n = 19)	ePTFE (n = 10)	*p*
Age in week	13 (10–20)	12 (10–20)	0.25
Body weight, g	450 (360–510)	405 (350–710)	0.16
Surgical time, min	43 (37–61)	48 (42–53)	0.10

SF; silk fibroin; ePTFE, Expanded polytetrafluoroethylene.

### Graft patency

**Figure 3:**
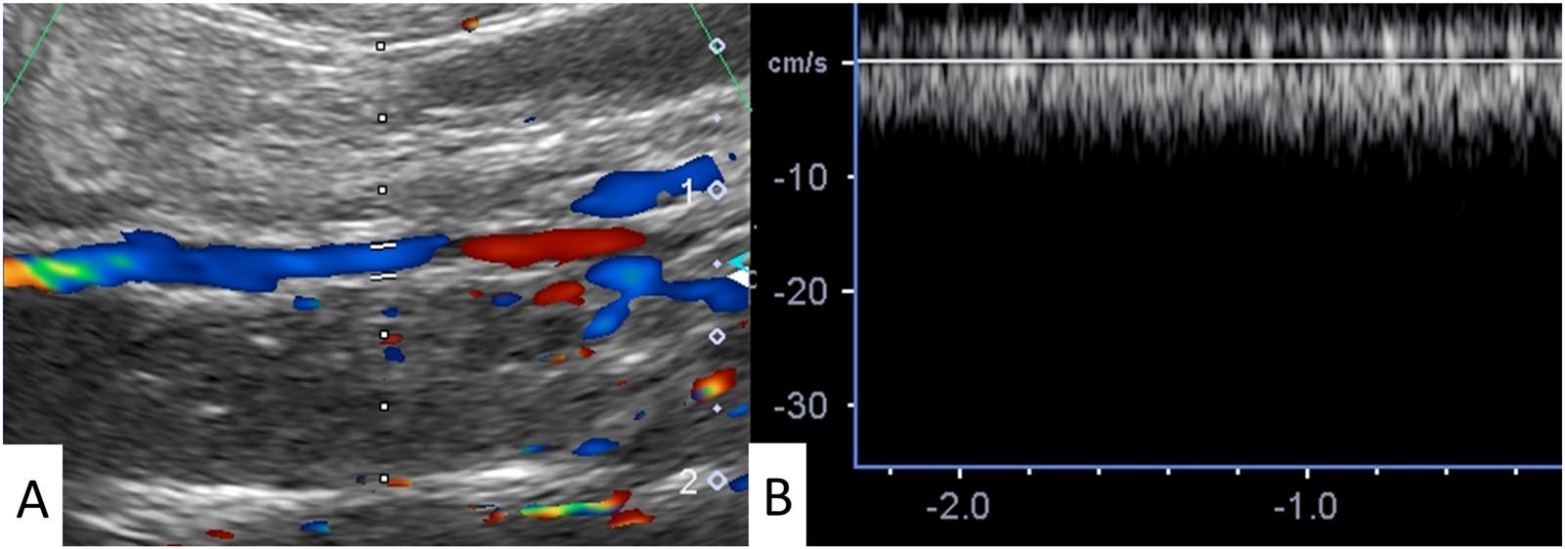
A representative color Doppler ultrasonography and Doppler flow imaging showed colored flow from the distal (**A**: right) to proximal sides (left) and steady shear flow (**B**) in the SF group. Images were processed by a software, Microsoft PowerPoint Ver. 365, https://www.microsoft.com/ja-jp/education/products/office.

**Figure 4:**
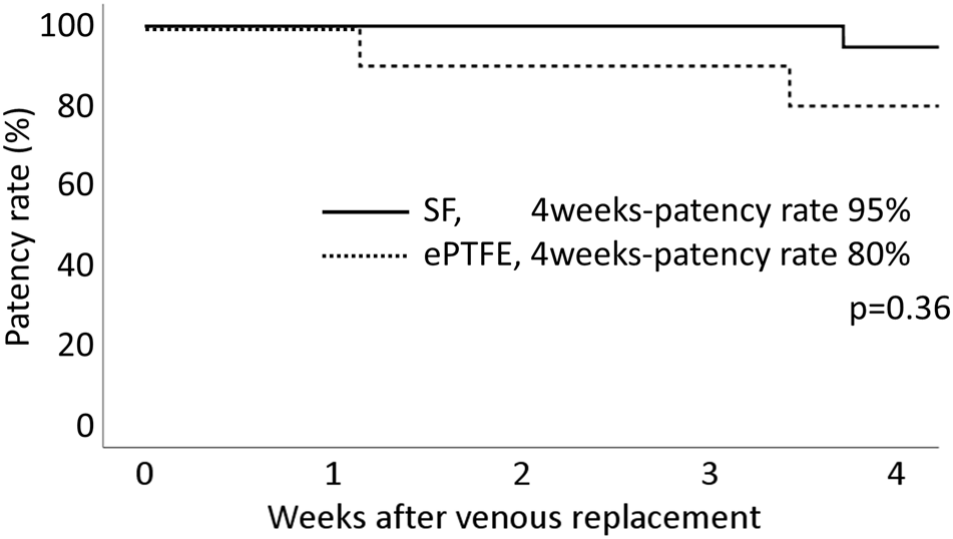
The patency rates at 1 and 4 weeks after replacement in the SF group (solid line) were 100.0% and 94.7%, respectively, whereas in the ePTFE group (dotted line), the patency rates were 100.0% and 80.0%, respectively. The difference between the 2 groups was not statistically significant (*p* = 0.36). ePTFE; Expanded polytetrafluoroethylene, SF; silk fibroin. An image was made by a software, IBM SPSS Statistics, Ver. 25.0, https://www.ibm.com/jp-ja/products/spss-statistics.

A representative Doppler ultrasonography result showing colored flow from the distal to proximal sides and ~ 5 cm/s steady shear flow in the SF group is shown in Fig. 3. The patency rate at 1 and 4 weeks after replacement in the SF group was 100.0% (n = 19/19, solid line) and 94.7% (18/19), respectively, whereas in the ePTFE group, it was 100.0% (10/10, dotted line) and 80.0% (8/10), respectively. The difference between groups was not significant (*p* = 0.36, Fig. 4). One SF vascular graft occluded at 3 weeks 5 days after implantation. The loss of patency was due to thrombosis because red blood cells and fibrin had accumulated inside the lumen.

### Histologic analysis

**Figure 5:**
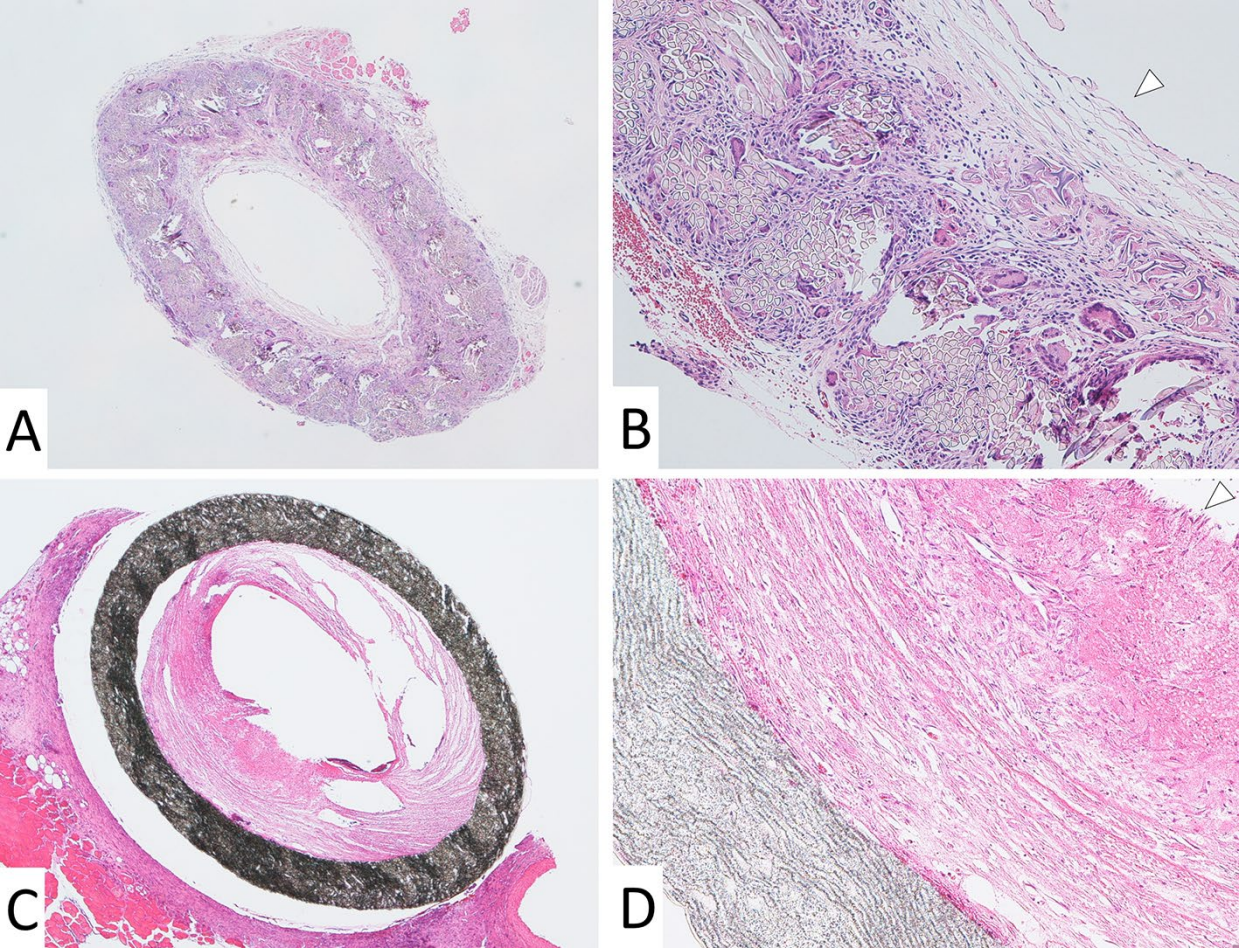
Microscopic findings of the SF vascular graft (**A** 40x, **B** 100x) and ePTFE vascular graft (**C** 40x, **D** 100x). The lumen of the SF vascular graft was fully patent (**A**), whereas the lumen of the ePTFE vascular graft remained patent, but was narrowed (**C**). In high-power micrographs, cellular components were observed between the SF fibers, and the luminal and outer surfaces of the SF vascular graft were covered with flat cells (**B**). Fibrin accumulation was observed on the luminal surface of the ePTFE vascular graft, but no flat cells were observed (**D**). ePTFE; Expanded polytetrafluoroethylene, SF; silk fibroin. Images were taken by a software, Nikon DS-L2, Ver. 4.61, https://www.nikon.com/products/microscope-solutions/support/download/software/camerasfor/ds_l2_v461u.htm.

Hematoxylin and eosin staining of each graft is shown in Fig. 5. The lumens of both the ePTFE and SF vascular grafts remained patent, but the lumen of the ePTFE graft was circumferentially narrowed (Fig. 5A,B). In the SF vascular graft, cellular proliferation was observed around the SF fibers, and the luminal and outer surfaces were covered by flat cells (Fig. 5B, arrowhead). In the ePTFE vascular graft, fibrin fibers accumulated, but no flat cells were observed on the luminal surface (Fig. 5D, arrowhead).

**Figure 6:**
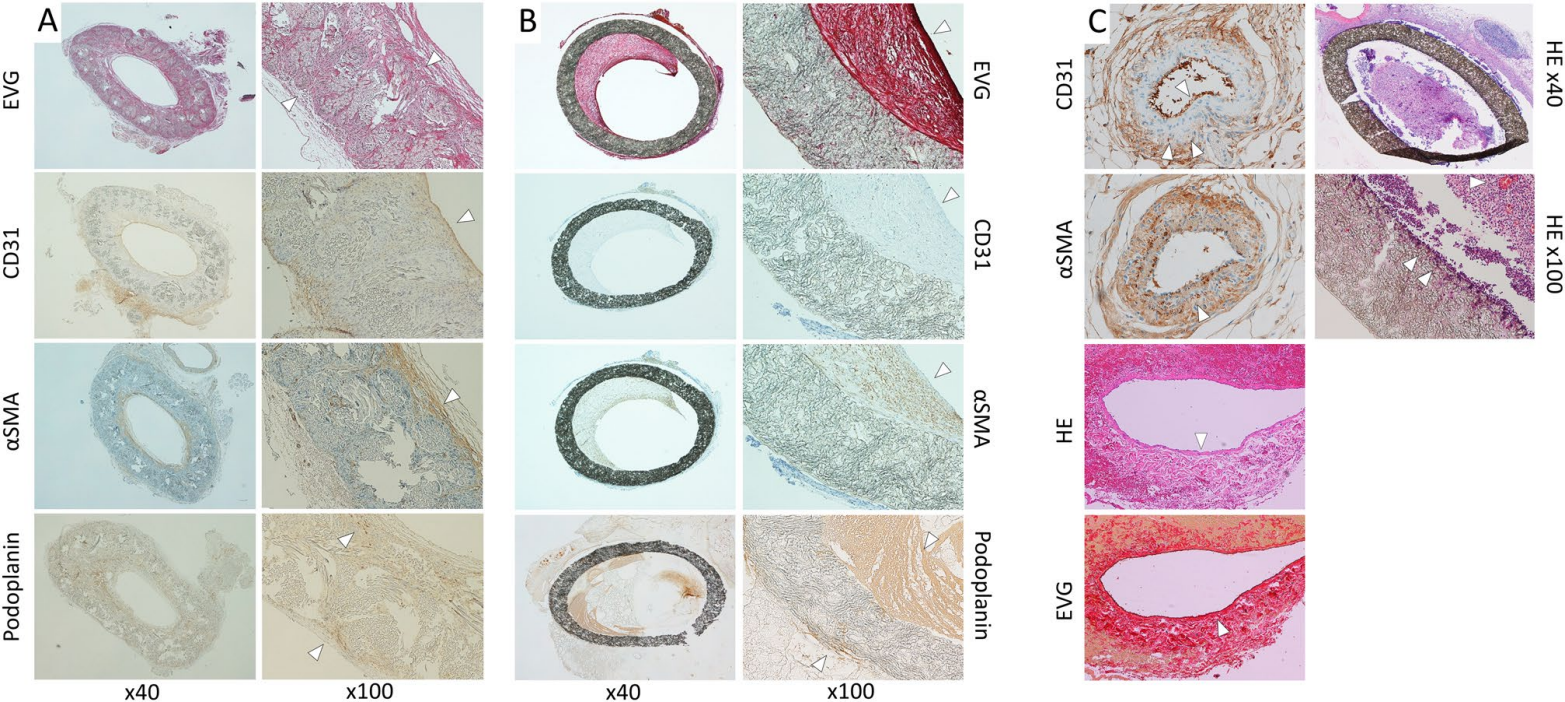
Elastica van Gieson staining of the SF graft revealed proliferation of the collagen fibers around the SF fibers, but no elastic fibers were observed (**A**, EVG, × 100, arrowhead). CD31 was expressed on the luminal surface of the SF vascular graft (**A**, CD31, arrowhead). αSMA antibody staining of the SF vascular graft was positive and backing CD31 positive cells (**A**, αSMA, arrowhead). Podoplanin was weakly expressed on the outer surface and partially in the wall of the SF vascular graft (**A**, Podoplanin, arrowhead). In the ePTFE graft, Elastica van Gieson staining showed thick fibrin inside the lumen (**B**, EVG, × 100, arrowhead). No CD31positive cells were observed on the luminal surface of the ePTFE vascular graft (**B**, CD31, arrowhead). Anti-αSMA antibody staining was positive inside the lumen in thick fibrin, which may indicate intimal thickening (**B**, αSMA, arrowhead). Podoplanin-positive cells were found not only inside the lumen, but weak positive staining was also observed on the outer surface of the ePTFE vascular graft (**B**, Podoplanin, arrowhead). As a positive control of the rat small artery, positive CD31 staining of endothelial cells was observed in an inner surface with a thin layer of lumen (**C**, CD31, single arrowhead). In the negative control of CD31, there was no CD31 staining in the thick smooth muscle layer (**C**, CD31, double arrowheads), in contrast to αSMA positive thick smooth muscle layer (**C**, αSMA, arrowhead). An example of native rat IVC showed lining endothelial cells at inner surface in HE staining (**C**, HE, arrowhead) and smooth muscle and collagen fibers layer around lumen in Elastica van Gieson staining (**C**, EVG, arrowhead). An occluded ePTFE vascular graft (**C**, HE × 40) at 3 weeks 3 days showed neutrophils infiltrated inside the graft aggregating around erythrocytes (**C**, HE × 100, single arrowhead) and lymphocytes (**C**, HE × 100, double arrowhead), indicating infection and inflammation, respectively. αSMA; α-smooth muscle actin, SF; silk fibroin, IVC; inferior vena cava, HE; Hematoxylin and eosin, ePTFE; expanded polytetrafluoroethylene. All images were taken by a software, Nikon DS-L2, Ver. 4.61, https://www.nikon.com/products/microscope-solutions/support/download/software/camerasfor/ds_l2_v461u.htm.

**Figure 7:**
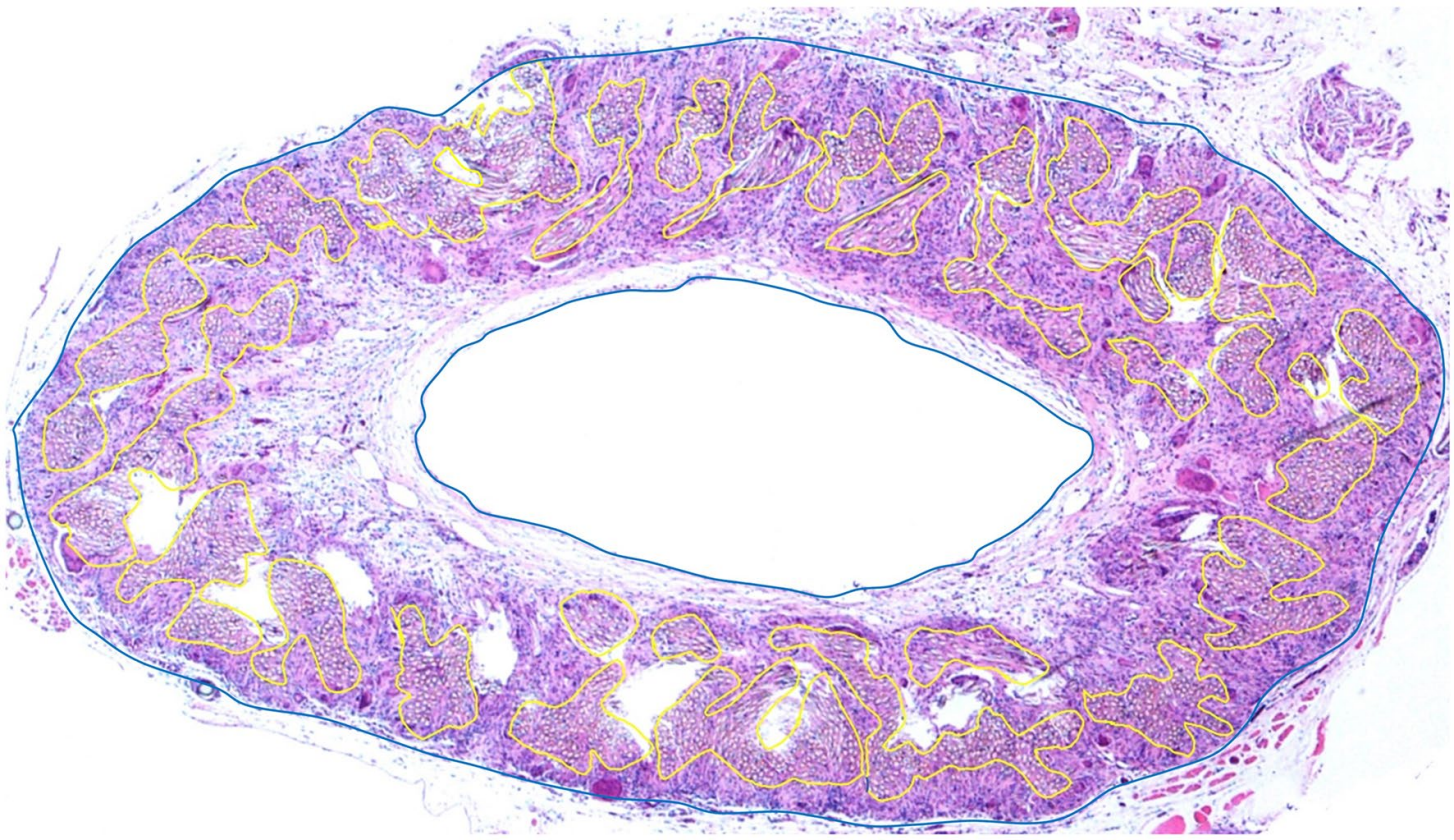
Semi-quantitative analysis using Image J^35^ was used to determine the ratio of the SF fibers and infiltrated tissue area of the representative cross-section of the SF graft wall. In one example of a cross section, the areas of the SF fibers and infiltrated native cells were calculated to be 48% and 52% of the total cross-sectional area of the SF vessel graft. SF; silk fibroin. An image was made by a software, Image J, Ver. 1.8.0_172., https://imagej.nih.gov/ij/index.html.

Elastica van Gieson staining of the SF vascular graft revealed collagen fibers, red, around the SF fibers, but no elastic fibers, black, (Fig. 6A, EVG, arrowhead). CD31 was expressed on the luminal surface of the SF vascular graft (Fig. 6A, CD31, arrowhead). Anti- alpha smooth muscle actin (αSMA) antibody staining of the SF vascular graft was positive and backing CD31 positive cells (Fig. 6A, αSMA, arrowhead). Podoplanin was weakly expressed on the outer surface and partially in the wall of the SF vascular graft (Fig. 6A, Podoplanin, arrowhead). The SF vessel graft wall was filled with collagen fibers. In one example of a cross section, the areas of the SF fibers and infiltrated native cells were calculated to be 48% and 52% of the total cross-sectional area of the SF vessel graft (Fig. 7).

In the ePTFE graft, Elastica van Gieson staining showed thick fibrin inside the lumen (Fig. 6B, EVG, arrowhead). No CD31 positive cells were observed on the luminal surface of the ePTFE vascular graft (Fig. 6B, CD31, arrowhead). Anti-αSMA antibody staining was positive inside the lumen in thick fibrin, which may indicate intimal thickening (Fig. 6B, αSMA, arrowhead). Podoplanin-positive cells were found not only inside the lumen, but weak positive staining was also observed on the outer surface of the ePTFE vascular graft (Fig. 6B, Podoplanin, arrowhead).

As a positive control of the rat small artery, positive CD31 staining of endothelial cells was observed in an inner surface with a thin layer of lumen (Fig. 6C, CD31, single arrowhead). In the negative control of CD31, there was no CD31 staining in the thick smooth muscle layer (Fig. 6C, CD31, double arrowheads), in contrast to αSMA positive thick smooth muscle layer (Fig. 6C, αSMA, arrowhead).

An example of native rat IVC showed lining endothelial cells at inner surface in Hematoxylin and eosin staining (Fig. 6C, HE, arrowhead) and smooth muscle and collagen fibers layer around lumen in Elastica van Gieson staining (Fig. 6C, EVG, arrowhead).

An occluded ePTFE vascular graft (Fig. 6C, HE × 40) at 3 weeks 3 days showed neutrophils infiltrated inside the graft aggregating around erythrocytes (Fig. 6C, HE × 100, single arrowhead) and lymphocytes (Fig. 6C, HE × 100, double arrowhead), indicating infection and inflammation, respectively.

## Discussion

This is the first report of venous replacement using an SF vascular graft in a rat model. We compared the patency rate and histologic reaction between SF vascular grafts and ePTFE grafts. The SF grafts had a favorable patency rate, and better endothelialization and collagen fiber infiltration than the ePTFE grafts at 4 weeks after venous replacement. SF may thus be an ideal scaffold material for grafting in the abdominal venous system, such as for replacing the IVC.

In present study, the SF graft had a 95% patency rate at 4 weeks, whereas the ePTFE graft had an 80% patency rate, although the difference was not statistically significant. Unlike reports on arterial replacement, there are few reports of animal models of abdominal venous replacement. In a canine IVC replacement model, an ePTFE graft had a 100% patency rate at 4 weeks, but more than half of each ePTFE graft lumen was filled with a thrombus^20^. Several studies have reported the patency rate of venous replacements in the clinical setting. For hepatic vein replacement, the 4-week patency rate of autologous vein grafts is 100%, whereas that of cryopreserved homologous veins is 95%^10^. In contrast, the 4-week patency rates of ePTFE grafts is only 81%^21^. Our findings revealed similar patency rates between SF and ePTFE grafts. Moreover, the long-term 1-year patency rates of autologous and cryopreserved homologous veins are 68% and 55%, respectively, for hepatic vein replacement^10^. The 4-month patency rate of ePTFE grafts is 39% and ePTFE grafts tend to occlude earlier^21^. As we only observed graft patency for a relatively short period ( 4 weeks ), additional studies are needed to evaluate the long-term patency rate of SF grafts for venous replacement in an animal model.

The remodeling processes is key to developing tissue-engineered vascular graft using various scaffold materials. Of these, SF is a biodegradable protein derived from silk that provides an ideal scaffold for various cell types in tissue engineering because of their favorable biocompatibility^22^. Endothelialization of remodeling processes contributes antithrombogenic and antiatherogenic properties for better patency^23^, and was reported for an SF vascular graft model of artery replacement^16^. Endothelial cells covered most of the luminal surface of the SF graft walls at 12 weeks after artery replacement^18^. Haga and colleagues reported the early completion of endothelialization at 3 weeks after rat aorta replacement with an SF graft^16^. Furthermore, endothelialization protects the graft from infection^23^, but endothelialization is not observed in ePTFE grafts^24^. In present study, CD31-positive endothelial cells covered the whole luminal surface of the SF vascular grafts at 4 weeks. These findings suggest the potential application of the SF vascular graft for abdominal venous replacement in a contaminated surgical field, such as in hepato-biliary-pancreatic surgery.

ePTFE vascular grafts cannot absorb blood because the ePTFE membrane is resistant to liquid infiltration^24^. Although SF fibers are coated with glycerin to prevent blood leakage between the fibers during replacement^25^, in the present study, the SF vascular grafts quickly absorbed blood after recanalization without bleeding (Fig. 1C). This absorption ability may make SF fibers biocompatible. According to several reports, SF fibers provide a durable scaffold as a vessel wall, but they are degradable, decompose in vivo, and are absorbed slowly^26^. In an artery replacement model, the amount of collagen tissue fibers increased while the amount of SF fibers decreased^18^. We observed collagen fiber proliferation around SF fibers. This may also contribute to their biologic compatibility^27,28^, and sufficient collagen accumulation may enhance the strength as a framework of the vessel wall^18^. Additionally, in the present study, some αSMA positive cells were observed, indicating that smooth muscle cells become established on the SF vascular graft^29^. Although this remodeling process corresponds to the artery replacement SF graft model, it seemed to be thinner than the artery^18^.

In the present study, podoplanin-positive cells were observed on the outer surface of the SF vascular graft, indicating that mesothelial cells were established. Podoplanin is a mesothelial marker^30^ and outer side of rat native IVC is covered by mesothelium (retroperitoneum) in general. If the outer surface of the SF graft was framed by mesothelial-like cells, the SF graft would have tolerance against infection because mesothelial cells may provide a non-adhesive and protective abilities for the replacement SF graft^31^. In the present study, however, the inside wall of the SF and the fibrin-like tissue of the ePTFE graft also showed podoplanin staining. The significance of podoplanin for venous remodeling in the present experiment model requires further investigation.

Although the wall structure of the rat native aorta is similar to that of the inferior vena cava, the elastic fibers and smooth muscle are thicker^32^. Endothelialization and cell infiltration were similar in the SF vascular graft compared with a previously reported artery replacement model^18^, but the thickness of the elastic fibers and smooth muscle, and the remodeling speed may differ. Further studies are needed to elucidate the differences.

A limitation of the present study is that we did not assess infection tolerability and inflammatory response to the SF graft. Further experiments with not only a 4-week time-point, but also a shorter and longer observation periods are needed to clarify the remodeling processes, the infection tolerability and the differences between venous and artery replacement animal models.

In conclusion, SF vascular grafts may be a promising tissue-engineered scaffold material for replacement in the abdominal venous system.

## Methods

### SF vascular grafts coated with an SF sponge

The SF double-raschel knit tube made of *Bombyx mori* SF threads was prepared using a double needle bar raschel with ten 30-guage needles and 20 courses on a computer-controlled double-raschel knit machine (Fukui Wrap Knitting Co. Ltd. Fukui, Japan). In our previous report^33^, the permeability of SF grafts with a double bar-cord knitting pattern based on the international organization for standardization (ISO) 7198 procedure was 4 ml/cm^2^/min and its density was 10.96 mg/cm^2^. The double bar-cord knitting pattern produced by a double-raschel machine has better strength to avoid coming apart at the seams and a thicker structure to allow cell infiltration inside the knitting pattern as a scaffold.

The inner diameter of the SF tube was 3 mm. To remove silk sericin, the knitted SF tube was degummed in a mixture of sodium carbonate (0.08% w/v) and Marseille soap (0.12% w/v) solution at 95 °C for 2 h, as previously reported^19,33^. To make the SF sponge, an aqueous solution of SF was prepared. The remaining degummed SF fibers were dissolved by adding CaCl2-H2O-EtOH solution (molar ratio: 1:8:2) to SF at a concentration of 10% w/v and boiling them at 70 °C for 1 h. This solution was filtered to remove residual solid components, and then dialyzed against a cellulose dialysis membrane (36/32, MWCO 14,000, Viskase Companies, Inc., Lombard, IL, USA) at 4 °C for 3 days. An approximately 4% (w/v) SF aqueous solution was obtained and centrifuged for 30 min at 4 °C at 18,000 rpm to remove impurities. The knitted SF tube was then coated with the SF sponge was as follows. The SF tube was immersed into a pipe filled with the mixed aqueous solution at a ratio of 1 : 1 (w/w) SF and glycerin, which was used as a porogen for coating. The pipe was placed in a desiccator where the interior was kept under reduced pressure of 100 hPa, and the SF graft was removed from desiccator and frozen at − 20 °C overnight. The graft was then immersed in distilled water for 3 days with several water changes to remove the glycerin. The graft was placed a bag with distilled water and sterilized in an autoclave at 120 °C for 20 min.

### Scanning electron microscopy observation and mechanical properties of SF grafts

The morphology of the outer surface and the cross-section of the knit SF grafts coated with SF sponges were observed by scanning electron microscopy (VE-7800, Keyence Corp., Osaka, Japan).

The longitudinal suture retention strength, circumferential tensile strength, and circumferential compressive elastic modulus of the SF grafts coated with SF sponges were determined using a tensile testing machine, table-top material tester (EZ Graph , Shimadzu Corp., Kyoto, Japan) according to the previously reported methods^18,19,33,34^ . For the longitudinal suture retention test, a sample tube was cut to a length of 20 mm and then the suture was passed through 2 mm from the end., pulled by a clamp (3 mm/min.) using a 100 N operator cell until the breaking point, and analyzed. To determine the circumferential tensile strength and circumferential compressive elastic modulus, we prepared dry, short, ring-shaped specimens with an axial length of 10 mm for each tube. The load cell was set to 5 N, and the rate of stretching was 2 mm/min. The tensile strength was measured as a function of the stroke distance and the circumferential compressive elastic modulus was measured when the specimen was compressed using 10% of the inner diameter. All specimens were hydrated for 1 h before testing. Porosity, ε (%), was calculated by following the formula, ε (%) = (V2 − V1)/(V3 − V1) × 100. An SF graft was immersed in hexane solution (V1) for 10 min (hexane with SF Graft volume, V2) under reduced pressure (0.05 MPa). The volume of the hexane solution (V3) was measured again after removing the SF graft filled with hexane.

### Animal model

The study protocol (I-P16-034) was approved by the University of Tokyo Animal Ethics Committee in accordance with the Japanese and ARRIVE (Animal Research: Reporting of In Vivo Experiments) guidelines. Male Sprague–Dawley rats (CLEA Japan, Inc., Tokyo, Japan) were used. All rats were kept for 1 to 4 weeks in micro-isolator cages with a 12-h light/dark cycle and fed a certified diet (CRF-1, Charles River Laboratories Japan, Inc., Yokohama, Japan). The rats were fasted overnight before undergoing the surgical procedure.

### Surgical procedure

All surgical procedures were performed by hepato-biliary-pancreatic surgeons (S.K., D.I.). The rats were anesthetized by intraperitoneal injection of pentobarbital (30 mg/kg body weight). The IVC was exposed from the left renal vein at the cranial side to the bifurcation on the caudal side, and all branches of the IVC were ligated and divided with 6-0 nylon (Keisei Medical Industrial CO., LTD., Tokyo, Japan) knotted sutures using an electric scalpel. After intravenous injection of unfractionated heparin (100 IU/kg), the proximal and distal portions of the infra-renal IVC were clamped with vascular clips. A 10-mm segment of the IVC was removed and replaced with an SF vascular graft (10 mm long, 3 mm in diameter, n = 19 ) by end-to-end anastomosis using 10-0 nylon (Keisei Med.) sutures, starting with 2 stay sutures at 180° apart at both the cranial and caudal sides, then suturing the front wall, followed by the back wall. The cranial and caudal side anastomosis each required 12 stitches. The caudal and cranial sides of the vascular clamps were slowly removed, and vessel flow was restored through the SF vascular graft. Graft patency was confirmed by gross inspection. In another group of rats, the IVC was replaced with an ePTFE graft (10 mm long, 3 mm in diameter, W.L. Gore & Associates, Inc., Newark, DE, USA, n = 10 ) in the same manner. No anticoagulant, antiplatelet, or antibiotic agents were administered postoperatively.

### Patency assessment

The patency of the grafts in the SF and ePTFE groups was monitored by Doppler ultrasonography with a diagnostic ultrasound system (8-MHz sector probe, TUS-A300, Toshiba Corp., Tokyo, Japan) at 1 day and every 3 days for 1 to 4 weeks after venous replacement. Graft occlusion was defined as the absence of a color Doppler signal. When we found the absence of a color Doppler signal, the rat was anesthetized with an intraperitoneal injection of 30 mg/kg body weight pentobarbital and the graft was grossly and pathologically evaluated.

### Histologic analysis

At 4-weeks after replacement, rats underwent a general physical examination to evaluate their condition. Following induction of general anesthesia with an intraperitoneal injection of pentobarbital (50 mg/kg body weight), the rats were perfused with 0.9% saline solution through the left ventricle. The grafts were carefully removed together with the surrounding tissue. Tissue cross-sections were prepared at the middle of the SF and ePTFE vascular grafts and fixed in 10% formalin and snap-frozen in Tissue Tek O.C.T. compound (Sakura Finetek Japan Co., Ltd., Tokyo, Japan) for histologic analysis. The tissues were fixed in 10% formalin, embedded in paraffin, sectioned (4-µm thick, Tissue-Tek Auto Section), and then processed for hematoxylin and eosin staining. Elastica van Gieson stain was also applied to detect elastic and collagen fibers.

Semi-quantitative analysis was used to determine the ratio of the SF fibers and infiltrated tissue area of the representative cross-section of the SF graft wall. SF fibers, seen as aggregations of transparent dots on a representative cut surface were encircled by yellow lines on a histologic image. At the same time, infiltrated native cells, the other area of the whole SF graft wall, was encircled with a blue line. The ratio of the remaining SF fiber area (yellow) to native cells (blue) was determined to calculate the area of one cut surface of the SF graft that was replaced by native cells at 4 weeks. The analysis was performed using Image J software (version 1.44; National Institute of Mental Health Bethesda, MD, USA)^35^.

Immunohistochemical staining was performed as previously reported^29^. The sections were incubated with primary antibodies, including alkaline phosphatase-conjugated anti-αSMA (clone 1A4, MilliporeSigma, St. Louis, MO, USA), anti-rat CD 31 antibody (clone TLD-3A12, BD Biosciences, San Jose, CA, USA), and anti-podoplanin antibody (ab11936, Abcam, Cambridge, MA, USA) followed by incubation with biotinylated anti-mouse immunoglobulin (Ig) G secondary antibody (DAKO, Glostrup, Denmark). Histologic analysis was performed by one pathologist (M. T. ) without knowledge of the patency outcome. All pathological figures were made using Digital Sight camera control unit with software (Nikon DS-L2, Ver. 4.61, Nikon Corp., Tokyo, Japan, https://www.nikon.com/products/microscope-solutions/support/download/software/camerasfor/ds_l2_v461u.htm).

### Statistical analysis

Factors of both groups were compared using the Mann–Whitney U test. The log rank test was used to assess the patency rate in both groups. A *p*-value less than 0.05 was considered statistically significant. IBM SPSS Statistics for Windows, version 25.0 (IBM Corp, Armonk, NY, USA) was used for the analysis.

## Data Availability

The authors declare that the data supporting the findings of this study are available within the paper.
